# Type II ZnO-MoS_2_ Heterostructure-Based Self-Powered UV-MIR Ultra-Broadband p-n Photodetectors

**DOI:** 10.3390/molecules30051063

**Published:** 2025-02-26

**Authors:** Badi Zhou, Xiaoyan Peng, Jin Chu, Carlos Malca, Liz Diaz, Andrew F. Zhou, Peter X. Feng

**Affiliations:** 1 Department of Chemistry, Biochemistry, Physics, and Engineering, Indiana University of Pennsylvania, Indiana, PA 15705, USA; badi.zhou@pm.me; 2 College of Artificial Intelligence, Southwest University, Chongqing 400715, China; pengxy2015@swu.edu.cn (X.P.); chujin@swu.edu.cn (J.C.); 3 Department of Chemistry, University of Puerto Rico, San Juan, PR 00936, USA; carlos.malca@upr.edu (C.M.); liz.diaz2@upr.edu (L.D.); 4 Department of Physics, University of Puerto Rico, San Juan, PR 00936, USA

**Keywords:** two-dimensional materials, transition-metal dichalcogenides, photonics, ZnO-MoS_2_ heterostructure, p-n heterojunction, photovoltaic mode, photoconduction mode, ultra-broadband photodetectors, UV-MIR spectral range, vacancy defects, bandgap tuning, Internet of Things (IoT) sensing

## Abstract

This study presents the fabrication and characterization of ZnO-MoS_2_ heterostructure-based ultra-broadband photodetectors capable of operating across the ultraviolet (UV) to mid-infrared (MIR) spectral range (365 nm–10 μm). The p-n heterojunction was synthesized via RF magnetron sputtering and spin coating, followed by annealing. Structural and optical analyses confirmed their enhanced light absorption, efficient charge separation, and strong built-in electric field. The photodetectors exhibited light-controlled hysteresis in their I-V characteristics, attributed to charge trapping and interfacial effects, which could enable applications in optical memory and neuromorphic computing. The devices operated self-powered, with a peak responsivity at 940 nm, which increased significantly under an applied bias. The response and recovery times were measured at approximately 100 ms, demonstrating their fast operation. Density functional theory (DFT) simulations confirmed the type II band alignment, with a tunable bandgap that was reduced to 0.20 eV with Mo vacancies, extending the detection range. The ZnO-MoS_2_ heterostructure’s broad spectral response, fast operation, and defect-engineered bandgap tunability highlight its potential for imaging, environmental monitoring, and IoT sensing. This work provides a cost-effective strategy for developing high-performance, ultra-broadband, flexible photodetectors, paving the way for advancements in optoelectronics and sensing technologies.

## 1. Introduction

Photodetectors play a vital role in modern optoelectronic systems by converting light into electrical signals, enabling applications in optical communication, imaging, environmental monitoring, medical diagnostics, and real-time Internet of Things (IoT) sensing [1]. The increasing demand for high-performance photodetectors with broadband sensitivity, capable of detecting light from the ultraviolet (UV) to the infrared (IR) and terahertz (THz) regions, has driven extensive research into novel materials and advanced device architectures. Among the wide array of broadband photodetectors, molybdenum disulfide (MoS_2_)-based devices have emerged as highly promising due to their unique optoelectronic properties, including a tunable bandgap, high carrier mobility, and strong light–matter interaction [2]. Recent advancements in MoS_2_-based photodetectors have focused on leveraging heterostructures, doping, and plasmonic enhancement to extend their detection capabilities across a broad spectral range, enhancing their sensitivity and response times. Integrating MoS_2_ with other two-dimensional (2D) materials or nanostructures has led to devices with an improved performance, as summarized in Table 1. However, challenges remain in achieving a wide spectral response, high responsivity, fast response times, and low power consumption [3,4,5].

A key strategy for addressing these challenges is the development of heterostructure-based photodetectors that exploit the complementary properties of different materials through bandgap engineering [6]. Although ZnO is a wide-bandgap semiconductor with strong UV absorption only while MoS_2_ is sensitive to visible light, theoretical studies have shown that vacancies and defects in ZnO-MoS_2_ heterostructures can significantly alter their electronic and optical properties, possibly enabling efficient broadband absorption up to the THz range [7,8,9]. Despite these predicted advantages, ZnO-MoS_2_ heterostructure-based broadband photodetectors have scarcely been tested. Apart from theoretical studies, further efforts are needed to investigate the fabrication methods and characterize the performance of ZnO-MoS_2_ heterostructure-based broadband photodetectors, with a focus on addressing key performance limitations in the spectral response range.

**Table 1:** State-of-the-art performance of MoS_2_-based broadband photodetectors.

Active Material	Wavelength λ (μm)	Bias Voltage (V)	Responsivity (mA/W)	Response Time ^1^ (ms)	Ref.
MoS_2_	0.4–1.1		1.4 × 10^7^ (@700 nm)	2.5	[10]
MoS_2_	0.445–2.717		50.7		[11]
MoS_2_ + Si	0.405–0.98	1 V	74.6	t_r_ = 0.178, t_d_ = 0.198	[12]
MoS_2_ 2D/0D	0.365–0.78		12.8 (@554 nm)		[13]
MoS_2_ + ZnS	0.365–0.78		0.0179 (@554 nm)		[14]
MoS_2_ + CuInSe_2_-QDs	0.355–1.064	5 V	74.8 × 10^3^ (@1064 nm)		[15]
MoS_2_ + PDPP3T	0.38–0.98		12.4 (@380 nm)		[16]
MoS_2_ + Ga_2_O_3_	0.254–1.2	5 V	171 × 10^3^ (@900 nm)	t_r_ = 0.097, t_d_ = 0.114	[17]
MoS_2_ + P(VDF-TrFE)	0.375–10	5 V	140 (@2.76–10 μm)	5.5 (@2.76–10 μm)	[18]
MoS_2_ + V_2_O_5_	0.365–0.78	−1 V	65.1 (@554 nm)	~40,000 (@554 nm)	[19]
MoS_2_ + SnS_2_-QD	0.365–0.78	1 V	435 × 10^3^ (@554 nm)	t_r_ = ~100	[20]
MoS_2_ + CdS	0.365–0.7	1 V	5.5 × 10^3^ (@700 nm)	t_r_ = 100 (@610 nm)	[21]
Graphene + MoS_2_ + graphene	0.405–2	−1 V	414 × 10^3^ (@532 nm) 376 × 10^3^ (@2 μm)	692.5 (@532 nm)	[22]
ZnO + MoS_2_	0.365–10	0 V 0.5 V	0.17 (@940 nm) 1.46 (@940 nm)	t_r_ = 140, t_d_ = 100	This work

^1^ t_r_ = rise time; t_d_ = decay time.

This paper describes the fabrication of ZnO-MoS_2_ heterostructure-based broadband photodetectors through a two-step process aimed at enhancing MoS_2_’s optical absorption properties. ZnO films were first deposited onto a silicon wafer via RF magnetron sputtering, followed by the spin coating of MoS_2_ nanosheets. Multiple MoS_2_ layers were deposited to ensure a uniform distribution, and the heterostructures were annealed to improve the integration and material properties. Platinum electrodes were added to complete the photodetector prototypes, which featured a simple and cost-effective design. The devices were subsequently characterized to assess their performance, and the impact of Mo vacancies on the ZnO-MoS_2_ bilayer’s band structure was also simulated.

## 2. Properties of ZnO and MoS_2_ Heterostructures

**Figure 1:**
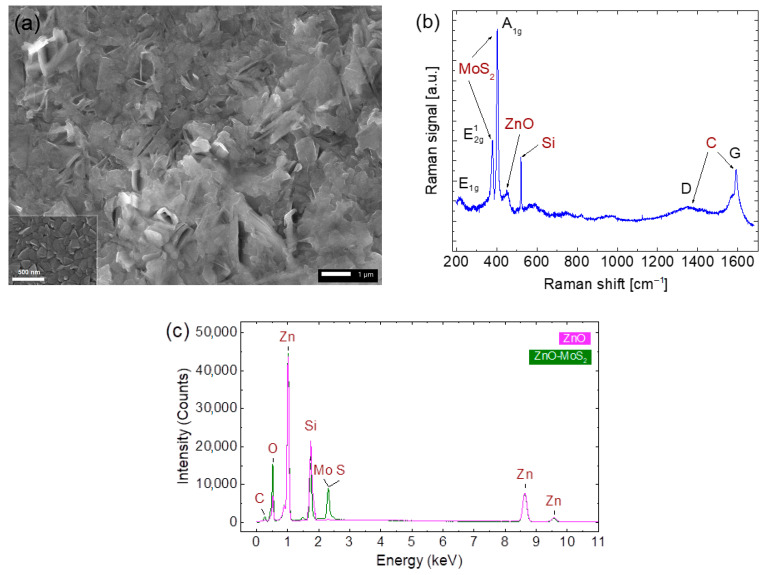
(**a**) SEM image of ZnO coated with MoS_2_, with a scale bar of 1 μm. Inset: ZnO nanoparticles; scale bar: 500 nm. (**b**) Raman spectrum of the ZnO-MoS_2_ sample. (**c**) EDX spectra of ZnO and ZnO-MoS_2_ after annealing.

As reported, ZnO nanostructures can be 0D, 1D, 2D, or 3D [23], depending on the growth techniques and conditions. Hence, the surface morphologies of the prepared ZnO and MoS_2_ samples were analyzed in their composition using a JEOL 6480LV scanning electron microscope (SEM) (JEOL, Tokyo, Japan) equipped with energy-dispersive X-ray spectroscopy (EDX). Figure 1a shows the SEM images of a ZnO sample before and after MoS_2_ spin coating. The synthesized ZnO nanoparticles (Figure 1a inset) have smooth surfaces and diameters of 100 nm. The layer’s thickness was estimated to be approximately 1 µm [24]. In contrast, the ZnO-MoS_2_ heterostructures exhibit larger diameters and rougher surfaces due to the MoS_2_ nanosheets coated onto the surface of the ZnO nanoparticles. An average continuous MoS_2_ sheet has an estimated thickness of ~5 nm and a lateral size of approximately 4 µm^2^, with the total MoS_2_ thickness reaching about 500 nm due to the stacking of multiple randomly oriented MoS_2_ clusters [25].

Micro-Raman scattering measurements were performed using a Jobin-Yvon T64000 Triplemate system (Horiba Jobin-Yvon, Edison, NJ, USA), with 514.5 nm (2.41 eV) radiation from a coherent argon laser as the excitation source. The scattered data were collected and processed using a liquid-nitrogen-cooled charge-coupled device (CCD) system, as shown in Figure 1b. The MoS_2_ layer exhibited characteristic MoS_2_ Raman modes at 379 and 402 cm^−1^ corresponding to the $E_{2g}^{1}$ and $A_{1g}$ modes, respectively, with an interval of 23 cm^−1^, which indicated that the MoS_2_ nanosheets were predominantly multilayer [26], while the peak at ~200 cm^−1^, marked as E_1g_, most likely corresponded to the in-plane vibrational mode of MoS_2_, activated due to the interlayer van der Waals interactions [27].

The Raman results show that the formation of the ZnO-MoS_2_ heterojunction led to both band broadening and band shifting compared to these values in the individual materials, indicating that the combination of MoS_2_ and ZnO in a heterostructure led to changes in the electronic structure and enhanced the photoelectric performance compared to that of single-material-based prototypes [28]. While defects and doping can influence the Raman spectra, no significant peak shifts or additional defect-related features were observed in our data, suggesting that these factors were not the primary contributors to the 200 cm^−1^ peak. However, we acknowledge the possibility of ZnS’s formation at the interface due to a chemical reaction between ZnO and MoS_2_, particularly during thermal annealing. The weak and broad nature of the ~200 cm^−1^ peak may be linked to ZnS, as reported in previous studies [29].

The Raman signal from the ZnO nanoparticles was weak and broad, attributed to the lower degree of crystallinity due to the room-temperature deposition, as well as the overcoating of the MoS_2_ layers. The peak at 520 cm^−1^, corresponding to the characteristic first-order phonon of the silicon from the substrate with a (100) crystal orientation [30], has been widely used as a reliable calibration of Raman measurements. As a comparison, when a Raman measurement was taken along the edge of the synthesized layers, there was no significant change, except a giant signal at this wavenumber being observed. The Raman spectroscopy analysis also confirms the presence of amorphous carbon, as indicated by the D band at 1350 cm^−1^ and the G band at 1580 cm^−1^, characteristic of sp^2^ hybridization.

Figure 1c exhibits the EDX mapping analysis of the ZnO sample before and after MoS_2_ coating and annealing. Prior to MoS_2_ deposition, the atomic percentage ratio of Zn to O was 42.5%/26.34% = 1.6, with additional signals from silicon (Si) originating from the substrate and from carbon (C) from contamination in the RF magnetron sputtering chamber. The observed Zn/O ratio indicates the presence of oxygen vacancies, which are common native point defects in ZnO. These vacancies contribute to the material’s intrinsic n-type conductivity by acting as positively charged donors. Following MoS_2_ coating and annealing, the EDX spectra reveal no significant change in the Zn signal, apart from the Mo and S signals overlapping at 2.3 keV. However, a notable increase in the C, O, and Si signals suggests additional contamination introduced during the annealing process.

An elemental analysis post-MoS_2_ deposition and annealing identified zinc (21 at%), oxygen (38 at%), molybdenum (0.96 at%), and sulfur (4.2 at%). The relatively low Mo and S content is attributed to the simple and cost-effective spin-coating process, which results in a thin MoS_2_ layer over the ZnO nanoparticles. While the synthesized ZnO nanoparticles exhibited intrinsic n-type conductivity with a high electron density [31,32], the Mo vacancies in the MoS_2_ induced p-type behavior [33]. These findings confirm the formation of a p-n heterojunction in the ZnO-MoS_2_ samples.

**Figure 2:**
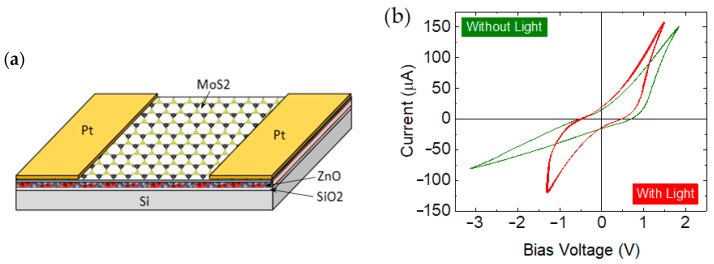
(**a**) Schematic of the ZnO-MoS_2_ heterostructure-based broadband photodetector. (**b**) The room-temperature current–voltage (I-V) characteristics without and with light illumination, corresponding to a dark current and a photocurrent (illuminated by 450 nm light with an intensity of 50 mW/cm^2^). Each hysteresis loop represents 10 repeated measurements.

Subsequently, Au electrodes were deposited using the plasma sputtering technique onto the two sides of the synthesized ZnO–MoS_2_ heterostructures to build prototypes. Figure 2a shows a schematic of a prototypic photodetector. The I-V properties were characterized, as shown in Figure 2b, using an HP34401 and a Keithley 6517A multimeter controlled via the LabVIEW 2024 Q3 program. The measurements were carried out in standard ambient conditions without and with light illumination. Stable hysteresis loops were observed when the heterostructure was operated at room temperature.

As shown in Figure 2b, the I-V curve exhibits the characteristic behavior of a simple p-n junction diode [34]. In the reverse bias region, a low reverse saturation current through the device indicates that the backward current is partially suppressed. In contrast, the forward current increases gradually at first and then rapidly with an increase in the forward voltage. It is interesting to note that while neither ZnO nor MoS_2_ is intrinsically ferroelectric, the synergistic effects within the heterostructure—arising from the piezoelectricity of ZnO and the vacancy defects in MoS_2_—may facilitate polarization switching under an external electric field.

In the absence of light illumination, the observed hysteresis suggests resistive switching due to the bilayer’s interfacial properties and the intrinsic defects in the ZnO-MoS_2_ bilayer. The non-zero open-circuit voltage (V_oc_) is measured at −0.56 V and 0.68 V, respectively, when the dark current is zero. Similarly, the short-circuit current (I_sc_) is −15 μA and 15 μA, respectively, at zero bias voltage. Under 50 mW/cm^2^ blue light (λ = 450 nm) irradiation, a light-controlled hysteresis loop emerges, demonstrating an increase in the photocurrent with a rising bias voltage due to photoconductivity. Preliminary investigations of V_oc_ and I_sc_ were conducted in our previous work [6], which were influenced by several factors like temperature, different light illumination, the active layer’s properties, the quality of the interconnections, etc. The operating temperature appears to have a significant impact on V_oc_, while light illumination directly affects I_sc_.

The changes in the hysteresis curve under light illumination are likely driven by photo-induced modifications in the electronic and charge transport properties of the ZnO-MoS_2_ bilayer. Photons with energy exceeding the bandgap of ZnO and MoS_2_ generate electron–hole pairs, leading to an increase in the carrier density and enhanced conductivity, which explains the higher current observed in the hysteresis curve. The increase in the current under the applied bias suggests that the photovoltaic effect contributes to the charge transport. Additionally, both ZnO and MoS_2_ contain abundant defect states, as indicated by the EDX measurements, which can trap the charges during I-V cycling. Under illumination, photon energy liberates these trapped charges, altering the overall charge transport dynamics and reducing the hysteresis loop area. Furthermore, the built-in electric field generated by the photovoltaic effect can facilitate the carrier transport, further minimizing hysteresis and modifying the loop shape. These I-V characteristics confirm the ZnO-MoS_2_ bilayer’s sensitivity to light, making it a promising candidate for photodetectors or memory elements.

**Figure 3:**
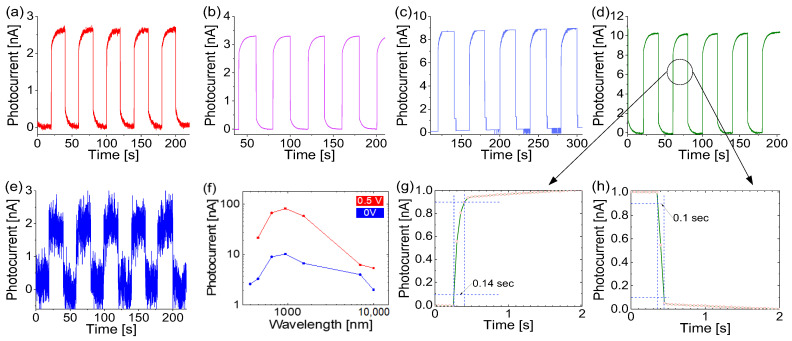
The measured photocurrent of the prototype when the device was operating at room temperature under 0 V bias, with on–off illumination light of different wavelengths at the same light intensity of 1.2 mW/cm^2^. (**a**) λ_UV_ = 365 nm, (**b**) λ_blue_ = 450 nm, (**c**) λ_red_ = 650 nm, (**d**) λ_NIR_ = 940 nm, and (**e**) λ_MIR_ = 10,000 nm. (**f**) The measured photocurrent under 0 V and 0.5 V bias as a function of the illumination wavelength. (**g**) The response time and (**h**) recovery time corresponding to 940 nm radiation.

Figure 3a–e present the measured time properties of the prototype when tested under light of various wavelengths ranging from 365 nm to 10 µm, all at a consistent intensity of 1.2 mW/cm^2^. The device was operated at room temperature under zero voltage bias, functioning as a self-powered photodetector. This capability minimizes the dependence on external power sources, enhancing its sustainability and making the device well suited to remote or inaccessible locations, aligning with the demands of IoT applications. However, at the 10 μm wavelength, the photocurrent exhibits a smaller signal with a significant noise background.

As shown in Figure 3f, the photodetector demonstrates a broadband wavelength response, with a photocurrent recorded under excitation wavelengths ranging from UV (365 nm) to MIR (10 μm), approximately 3.39 eV to 0.124 eV. The highest responsivity was observed at around 940 nm, reaching 0.17 mA/W at zero bias. In comparison, applying a 0.5 V bias increases the responsivity by a factor of eight, achieving 1.46 mA/W at 940 nm. These measurements suggest that the photodetector may have an even broader response extending into the THz range, though the experimental range validation was constrained by the available radiation sources. A higher bias voltage could further enhance its responsivity.

The photodetectors also have a fast photocurrent rise time (140 ms) and recovery time (100 ms), as shown in Figure 3g,h. The rise and recovery times follow the standard definition, i.e., the time intervals taken for a change from 0.1 to 0.9 in the normalized photocurrents, or vice versa. Also, the rise and recovery times were consistent under different wavelength illuminations, especially when the wavelength was further extended to 10 μm. This phenomenon indicates that the photocurrent should likely be generated from the same sensing mechanism, i.e., the possibility of thermal effects, such as a photothermoelectric effect, which could take a longer time, is ruled out [35]. The real response and recovery times might be shorter than those shown in Figure 3g,h due to the relatively large step size used in the measurements.

The relatively low measured responsivity arose from the poor crystallinity of the synthesized heterostructures. To enhance their crystallinity, high-temperature annealing was performed post-synthesis. Crystallinity significantly influences the minority carrier lifetime, which directly affects the device performance. Poor crystallinity introduces defects that shorten the minority carrier lifetime, reducing the responsivity. In contrast, an extended minority carrier lifetime increases the photocurrent but compromises the response speed. This intrinsic trade-off between the photocurrent gain and response time necessitates careful optimization to achieve a well-balanced performance.

When operating in photoconductive mode with a non-zero voltage bias, similar time characteristics to those in Figure 3 were observed but with larger photocurrent magnitudes at these wavelengths. Despite this similarity, the two mechanisms differ slightly: photoconductive mode generates a photocurrent when the light excites free carriers, whereas photovoltaic mode with zero voltage bias produces a photovoltage by separating the light-generated carriers through a built-in electric field. As the bias voltage increases, the response in photoconductive mode may become faster due to the enhanced carrier collection facilitated by the applied bias.

An increase in the photocurrent under blue light illumination has been observed in various nanomaterials and can arise from multiple factors, including localized surface plasmon resonance with metal nanoparticles [36], a ferro-pyro-phototronic effect under heating and cooling variations [37], photochemical reactions in nanofluidic devices [38], and a piezophototronic effect when the ambient pressure on the junction is increased to 23 MPa [39]. In the present study, while photoconductivity plays an important role, the observed increase in the photocurrent under light is also influenced by trap-limited processes and photovoltaic effects.

## 3. Discussion

Strain can induce bandgap narrowing in heterostructures; however, since our synthesis employed a room-temperature coating process, the induced strain is minimal compared to that in techniques such as molecular beam epitaxy (MBE) or chemical vapor deposition (CVD). Previous studies [40,41,42] have reported a redshift of approximately 70 meV per the percent of applied strain for direct transitions, implying an unrealistically high ~20% strain would be required for 10 µm MIR detection. Given the small lattice mismatch (~2%) between the hexagonal primitive unit cells of MoS_2_ and ZnO, along with the random orientation of the MoS_2_ nanosheets, the strain effects are expected to be minimal. This conclusion is further supported by previous characterizations using X-ray diffraction (XRD), X-ray photoelectron spectroscopy (XPS), and high-resolution transmission electron microscopy (HRTEM) [24,25,43]. Therefore, we primarily attribute the observed change in the bandgap to the Mo vacancies.

**Figure 4:**
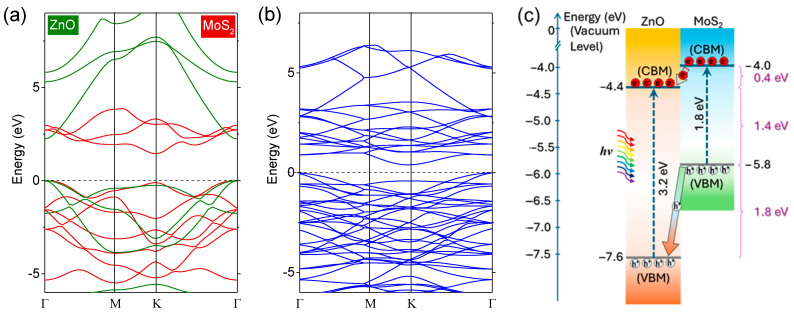
Simulated energy bandgaps of (**a**) ZnO and MoS_2_ monolayers and (**b**) ZnO-MoS_2_ heterostructure. The valence band maximum (VBM) is set to zero. (**c**) Schematic presentation of type II heterostructure formed of ZnO and MoS_2_. CBM: conduction band minimum.

**Figure 5:**
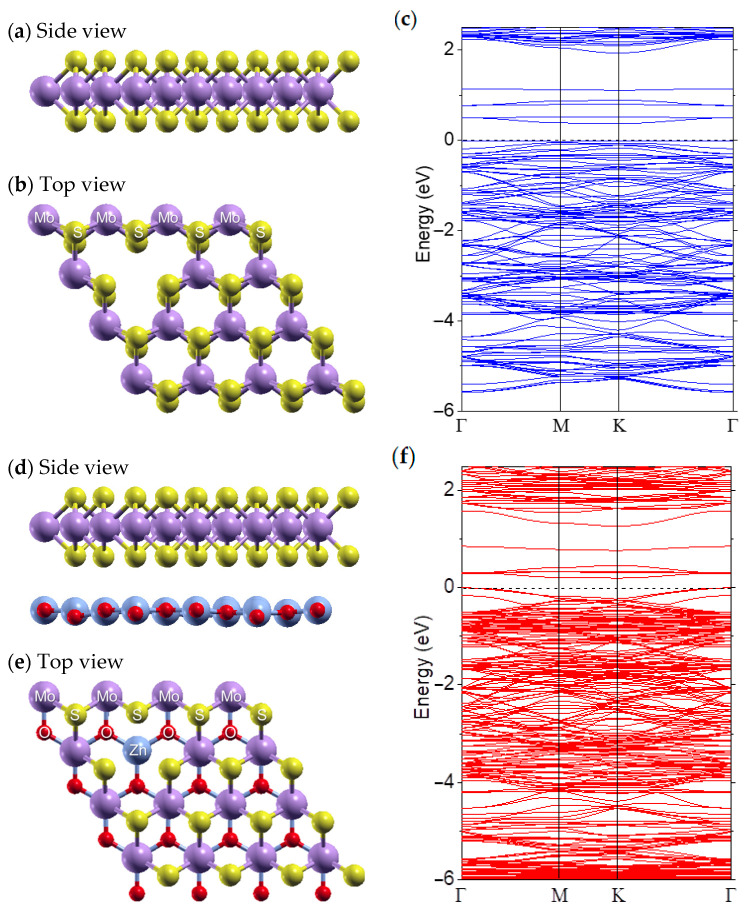
(**a**) Side view and (**b**) top view of the hexagonal-shaped wurtzite crystal structure of a ZnO monolayer with one Mo vacancy. (**c**) Band structure of MoS_2_ with one Mo vacancy. (**d**) Side view and (**e**) top view of a ZnO-MoS_2_ bilayer with one Mo vacancy. (**f**) Band structure of a ZnO-MoS_2_ bilayer with one Mo vacancy. The valence band maximum is set to zero.

As shown in Figure 4a, density functional theory (DFT) simulations confirm that the ZnO monolayer is a wide-bandgap semiconductor with a calculated bandgap of 3.2 eV, corresponding to a UV wavelength of 387 nm, while the MoS_2_ monolayer exhibits a bandgap of 1.8 eV, corresponding to a red wavelength of 689 nm. The band structure of the bilayer ZnO-MoS_2_ is presented in Figure 4b. The synthesized heterostructure exhibits a narrower bandgap of 1.4 eV, enabling the absorption to shift towards longer wavelengths of 886 nm in the NIR region. Furthermore, the transition to few-layer MoS_2_ results in a shift towards an indirect bandgap of approximately 1.3 eV, corresponding to a wavelength of ~954 nm [44]. The incorporation of MoS_2_ effectively narrows the bandgap of ZnO, broadening the absorption spectrum to span the UV-NIR range. For simplicity, we only consider the most stable stacking of the ZnO and MoS_2_ monolayers [45,46]. As shown in Figure 5d,e, the ZnO/MoS_2_ heterostructure adopts an energetically favorable stacking arrangement in which the Zn atoms are positioned directly above the Mo atoms, while O and S atoms reside at the hexagonal centers of the opposing layers.

First-principle calculations further reveal that the heterostructure exhibits van der Waals interactions at the interface [47]. A type II band alignment is observed in ZnO-MoS_2_ interfaces, facilitating effective charge separation driven by a built-in electric field, as illustrated in Figure 4c, where the bandgap data are based on the reported experimental values. The ZnO-MoS_2_ heterostructure demonstrates strong optical absorption across the UV, visible, and infrared regions, ultrafast carrier dynamics, enhanced charge transfer, and efficient photogenerated charge separation, making it highly suitable for photovoltaic and photodetector applications.

Introducing vacancy defects enables the bandgap of the ZnO-MoS_2_ heterostructure to be tailored to the sub-eV levels while maintaining the stability of the MoS_2_ [48]. Figure 5a,b illustrate a side view and a top view of the most stable hexagonal crystal structure of monolayer MoS_2_, comprising two sulfur atom layers sandwiching a molybdenum atom layer. The layers are bonded by strong covalent bonds. For simplicity, we only consider a single Mo vacancy, as depicted in Figure 5b. The corresponding band structure is shown in Figure 5c, revealing a bandgap of 0.43 eV, which corresponds to a wavelength of approximately ~2.9 µm.

DFT simulations indicate that Mo vacancies introduce shallow donor states near the conduction band (CB) and deep acceptor states near the valence band (VB), effectively narrowing the bandgap compared to that in pristine monolayer MoS_2_ without vacancies. The localized electronic states within the MoS_2_ bandgap arise from dangling bonds, altering the coordination environment around the vacancy site and enabling lower-energy (longer-wavelength) photon absorption. As a comparison, the calculated band structure of the ZnO-MoS_2_ bilayer (Figure 5f) with one Mo vacancy further confirms that the Fermi energy lies within the valence band, indicating the p-type property of MoS_2_ with Mo vacancies, and an even smaller bandgap of 0.20 eV is formed.

First-principle calculations by Yang et al. [48] show an interesting result—with an increase in the number of Mo vacancies, such as from single to double or triple vacancies, the bandgap narrows further, demonstrating that vacancy defect engineering is an effective approach to tuning the bandgap. At higher concentrations of Mo vacancies, the localized defect states overlap, forming impurity bands that can merge with the CB or the VB, resulting in significant bandgap narrowing. Mo vacancies can also lead to the formation of localized excitons with reduced binding energies, aligning with mid-infrared (MIR) photon energies.

Our experimental results align with the theoretical predictions, indicating that the Mo vacancies in MoS_2_ are critical for band structure engineering to enable mid-infrared radiation absorption at wavelengths of around 10 µm. By introducing localized defect states and impurity bands within the bandgap, the Mo vacancies effectively reduce the bandgap, facilitating lower-energy electronic transitions corresponding to the MIR photon energies. This defect-induced bandgap modulation is corroborated by both experimental data and theoretical models. As a result, controlled Mo vacancy introduction is a promising strategy for tailoring the optical and electronic properties of MoS_2_, broadening its potential for MIR-based technologies and advanced optoelectronic devices.

## 4. Materials and Methods

### 4.1. Photodetector Fabrication

Most ZnO and MoS_2_ binary heterostructures reported for broadband photodetectors are synthesized by depositing ZnO nanowires over MoS_2_ 2D nanosheets. In this paper, to emphasize the optical absorption properties of MoS_2_, we formed the heterostructure by coating ZnO with MoS_2_ through a two-step process. First, ZnO was deposited using RF magnetron sputtering. A silicon wafer with a 300 nm SiO_2_ insulating layer was used as the substrate, which was pre-cleaned through ultrasonic treatment in deionized water, ethanol, and acetone for 20 min. The ZnO films were deposited onto Si (100) substrates in a plasma sputtering deposition chamber, utilizing a commercial sintered ZnO target (99.9%, 2 inches, MTI Company, Richmond, CA, USA). The substrate-to-target distance was maintained at 7–8 cm. Deposition occurred at room temperature with an RF power of 200 W. The chamber’s base pressure was 10^−5^ Torr, and the working pressure, regulated by argon gas, was 8–10 mTorr.

Next, MoS_2_ was applied using spin coating. A commercially available dispersion of atomically thin MoS_2_ nanosheets (1 mg/mL in ethanol, XFNANO Materials Co., Ltd., Nanjing, China) was used. The thickness of the deposited layers was influenced by factors such as the solution’s viscosity, concentration, rotation speed, and the number of spin-coating cycles. To ensure a uniform distribution and minimize agglomeration, the MoS_2_ solution underwent ultrasonic cavitation for two hours prior to deposition. Each spin-coating step lasted 20 s at 3000 rpm. After each spin-coating cycle, the sample was placed in an oven at 80 °C for 5 min to dry before applying additional layers. Multiple layers were deposited to achieve a denser distribution of the MoS_2_ nanostructures on the ZnO’s surface. The ZnO-MoS_2_ heterostructures were then annealed in the air at 800 °C for two hours to enhance the material integration and improve the physical and chemical properties.

Finally, the ZnO–MoS_2_ heterostructure-based photodetectors were fabricated. Gold (Au) electrodes, 70–80 nm thick, were deposited on both sides of the active layer using plasma sputtering deposition. The fabricated prototype had an active layer exposure area of 10 × 0.5 mm^2^. The device’s structure was simple, and the fabrication process was rapid and cost-effective. Characterization and measurements of the photoconductor were conducted using a custom-built station [49].

### 4.2. The Computation Method

The first-principle calculations were carried out using the Quantum Espresso (QE) v.7.3 package [50], which is based on density functional theory (DFT) in a plane-wave basis set [51]. Density functional calculations utilizing generalized gradient approximation (GGA) of the Perdew–Burke–Ernzerhof (PBE) form to treat the exchange correlation contribution were performed to obtain the geometric structures [52]. For the monolayer calculations, the projected augmented wave (PAW) method was adopted to describe the pseudopotentials, and the valence configuration for the construction of the PAW potentials was Zn (3d^10^4s^2^), Mo (4s^2^4p^6^4d^4^5s^2^), O (2s^2^2p^4^), and S (3s^2^3p^4^). For bilayers with vacancies, norm-conserving (NC) pseudopotentials were used. Additionally, the DFT-D3 van der Waals (vdW) correction proposed by Grimme was utilized to describe weak vdW interactions [53], which improved the accuracy of the structural and electronic property calculations, aligning the theoretical bandgap values with the experimental results. As confirmed by the DFT calculations, the interfacial coupling in the Zno-MoS_2_ vdW heterostructures significantly impacts the device performance.

It is well known that DFT is notorious for underestimating bandgaps [54,55]. Hence, the Heyd–Scuseria–Ernzerhof (HSE06) hybrid density functional with a 25% Hartree–Fock exchange energy was used to obtain more accurate electronic structures [56]. For the monolayers, an energy cutoff of 800 eV, an energy convergence tolerance of 1.0 × 10^−8^ eV, convergence thresholds for the atomic force of 10^−2^ eV/Å, and a Monkhorst–Pack k-point grid of 13 × 13 × 1 for a 1 × 1 unit of the ZnO and MoS_2_ monolayers and 2 × 2 units of the ZnO-MoS_2_ systems were found to be sufficient for the geometrical optimization and electronic structure calculations. For bilayers with vacancies, an energy cutoff of 500 eV, an energy convergence tolerance of 1.0 × 10^−6^ eV, and a 5 × 5 × 1 k-point grid were used for 4 × 4 units of ZnO-MoS_2_. A thickness of the vacuum space of 20 Å between neighboring nanocomposites was adopted to avoid interactions. All of the structures were fully relaxed until the Hellmann–Feynman force on each atom was <0.01 eV/Å, leading to an interlayer spacing of 2.92 Å in the ZnO-MoS_2_ bilayer.

## 5. Conclusions

The type II heterostructure of ZnO and MoS_2_ reported in this study leverages the complementary properties of MoS_2_’s tunable bandgap and strong excitonic effects, along with ZnO’s UV sensitivity and light-trapping capabilities. The synergistic interaction between these two materials results in the ferroelectric-like property of the heterostructure, as well as a p-n heterojunction. With ZnO’s high electron mobility and high chemical stability and MoS_2_’s robust light–matter interaction over an ultrawide spectral range, the interface structure of the heterojunction further enhances the charge separation and reduces the recombination losses, thereby improving the overall device performance. Additionally, the formation of built-in electric fields at the heterojunction facilitates efficient charge transport, which is crucial for achieving high responsivity and fast response times.

The fabricated ZnO-MoS_2_ photodetectors have demonstrated remarkable advancements, including enhanced light absorption over an ultrabroad spectral range of up to 10 μm, efficient charge transfer, and fast response times. Under photovoltaic mode, self-powered devices based on this heterostructure exhibit excellent sensitivity across the UV, visible, and MIR regions, highlighting their potential for diverse applications in optical communication, imaging systems, and environmental sensing, even though the photodetector operates in both photovoltaic mode without bias and photoconductive mode with a bias voltage. Self-powered operation is ideal for real-time Internet of Things (IoT) applications and wearable electronics, complemented by the flexibility provided by the heterostructure obtained.

## Data Availability

The data presented in this study are available upon request.
